# Supplementary material for: Low Dietary Diversity and Low Haemoglobin Status in Ghanaian Female Boarding and Day Senior High School Students: A Cross-Sectional Study
Source: Medicina (Kaunas). 2024 Jun 26;60(7):1045. doi: 10.3390/medicina60071045 (PMC11279379; doi:10.3390/medicina60071045)
Supplement: Supplementary file 1 [file medicina-60-01045-s001.zip › medicina-3036245-supplementary.pdf]

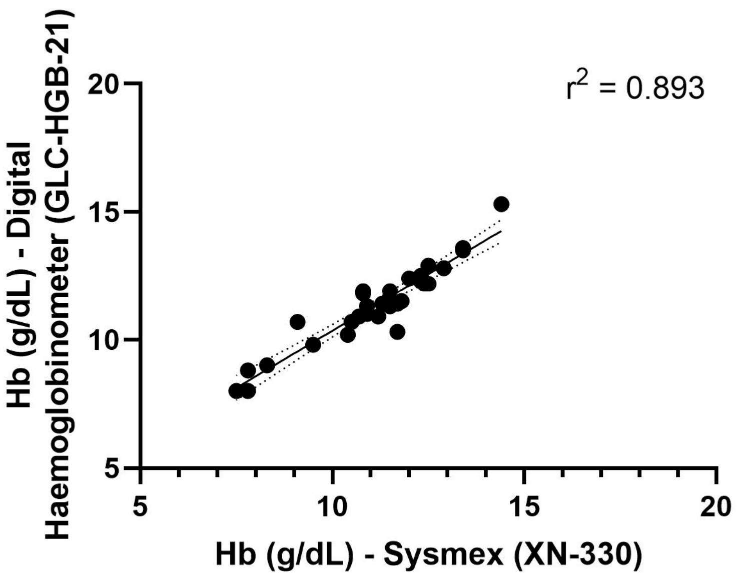

Figure S1. Validation of the digital haemoglobinometer with the laboratory-based measures

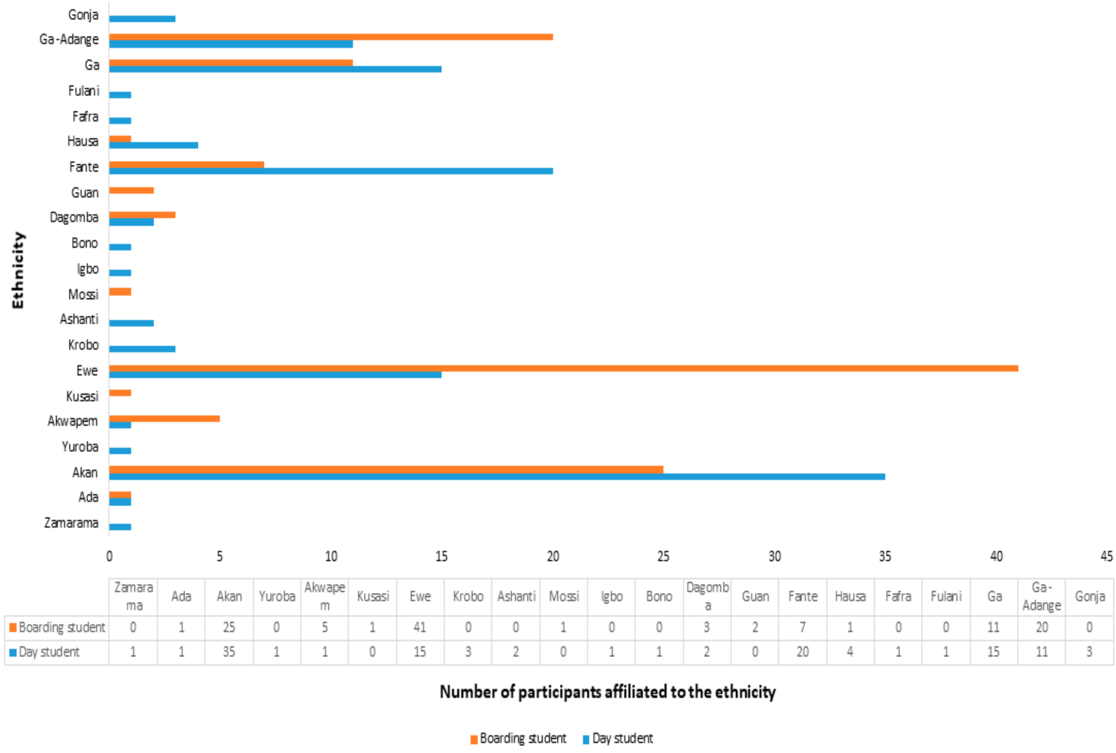

	Zamarama	Ada	Akan	Yuroba	Akwapem	Kusasi	Ewe	Krobo	Ashanti	Mossi	Igbo	Bono	Dagomba	Guan	Fante	Hausa	Fafra	Fulani	Ga	Ga-Adange	Gonja
Boarding student	0	1	25	0	5	1	41	0	0	1	0	0	3	2	7	1	0	0	11	20	0
Day student	1	1	35	1	1	0	15	3	2	0	1	1	2	0	20	4	1	1	15	11	3

Figure S2. Ethnicities of Boarding and Day adolescent students

Table S1. Categorisation of individual food items into ten food groups within the Minimum Dietary Diversity for Women (MDD-W)

Food groups	Examples of food
Grains, white roots and tubers, and plantains	Porridge, bread, rice, pasta/noodles, or other foods made from grains, millet, white potatoes, white yams, manioc/cassava/yucca, cocoyam, taro
Pulses (Beans, peas, and lentils)	Lentils, kidney, black eye beans, kidney beans, mature beans, or peas (fresh or dried seed), lentils or bean/pea products, koose
Nuts and seeds	Groundnut, tiger nuts, pumpkin seeds, almond, cashew and cocoa
Dairy	Milk, cheese, yogurt or other milk products
Meat, poultry and fish	Beef, pork, lamb, goat
Eggs	Eggs from chicken, duck, guinea fowl
Dark green leafy vegetables	Spinach, cocoyam leaves, dark green/leafy vegetables, including wild ones + locally available vitamin A-rich leaves such as amaranth, cassava leaves, kale, spinach
Other vitamin A-rich fruits and vegetables	Pawpaw, mango
Other vegetables	Okra, onion, beans (eaten as fresh pods)
Other fruits	Oranges, citrus juices, tangerine, melon
